# Police Encounters, Agitation, Diagnosis, and Employment Predict Psychiatric Hospitalisation of Intensive Home Treatment Patients During a Psychiatric Crisis

**DOI:** 10.3389/fpsyt.2021.602912

**Published:** 2021-02-05

**Authors:** Ansam Barakat, Matthijs Blankers, Jurgen E. Cornelis, Louk van der Post, Nick M. Lommerse, Aartjan T. F. Beekman, Jack J. M. Dekker

**Affiliations:** ^1^Department of Research, Arkin Institute for Mental Health Care, Amsterdam, Netherlands; ^2^Department of Psychiatry, Amsterdam University Medical Centres (UMC), Location VUmc, Amsterdam Public Health Research Institute Amsterdam UMC, Amsterdam, Netherlands; ^3^Trimbos-Institute, Netherlands Institute of Mental Health and Addiction, Utrecht, Netherlands; ^4^Department of Psychiatry, Amsterdam University Medical Centres (UMC), Location AMC, Amsterdam Public Health Research Institute Amsterdam UMC, Amsterdam, Netherlands; ^5^Department of Emergency Psychiatry, Arkin Institute for Mental Health Care, Amsterdam, Netherlands; ^6^Department of Research and Innovation, GGZ InGeest Specialized Mental Health Care, Amsterdam, Netherlands; ^7^Department of Clinical Psychology, Vrije Universiteit Amsterdam, Amsterdam Public Health Research Institute Amsterdam UMC, Amsterdam, Netherlands

**Keywords:** community mental health services, emergency psychiatry, intensive home treatment, randomised controlled trial, hospitalisation

## Abstract

**Objective:** This study aims to determine factors associated with psychiatric hospitalisation of patients treated for an acute psychiatric crisis who had access to intensive home treatment (IHT).

**Methods:** This study was performed using data from a randomised controlled trial. Interviews, digital health records and eight internationally validated questionnaires were used to collect data from patients on the verge of an acute psychiatric crisis enrolled from two mental health organisations. Thirty-eight factors were assigned to seven risk domains. The seven domains are “sociodemographic”, “social engagement”, “diagnosis and psychopathology”, “aggression”, “substance use”, “mental health services” and “quality of life”. Multiple logistic regression analysis (MLRA) was conducted to assess how much pseudo variance in hospitalisation these seven domains explained. Forward MLRA was used to identify individual risk factors associated with hospitalisation. Risks were expressed in terms of relative risk (RR) and absolute risk difference (ARD).

**Results:** Data from 183 participants were used. The mean age of the participants was 40.03 (SD 12.71), 57.4% was female, 78.9% was born in the Netherlands and 51.4% was employed. The range of explained variance for the domains related to “psychopathology and care” was between 0.34 and 0.08. The “aggression” domain explained the highest proportion (*R*^2^ = 0.34) of the variance in hospitalisation. “Quality of life” had the lowest explained proportion of variance (*R*^2^ = 0.05). The forward MLRA identified four predictive factors for hospitalisation: previous contact with the police or judiciary (OR = 7.55, 95% CI = 1.10–51.63; ARD = 0.24; RR = 1.47), agitation (OR = 2.80, 95% CI = 1.02–7.72; ARD = 0.22; RR = 1.36), schizophrenia spectrum and other psychotic disorders (OR = 22.22, 95% CI = 1.74–284.54; ARD = 0.31; RR = 1.50) and employment status (OR = 0.10, 95% CI = 0.01–0.63; ARD = −0.28; RR = 0.66).

**Conclusion:** IHT teams should be aware of patients who have histories of encounters with the police/judiciary or were agitated at outset of treatment. As those patients benefit less from IHT due to the higher risk of hospitalisation. Moreover, type of diagnoses and employment status play an important role in predicting hospitalisation.

## Introduction

There is consensus that psychiatric hospitalisation should be reduced in length or preferably prevented. There is a long history of efforts directed toward this goal. Intensive Home Treatment (IHT) is one of the more recent and better studied examples, and IHT is being implemented in many countries (1–4). The focus of IHT teams is on stabilising the psychiatric crisis in order to prevent hospitalisation and facilitate early hospital discharge by providing intensive care three times a week (5, 6). Earlier studies have found that IHT achieves a modest positive reduction of acute psychiatric hospitalisation and inpatient days (1, 7–10). Nevertheless, like any other treatment, IHT is no magic bullet and hospitalisation is not prevented in all patients. The identification of factors associated with psychiatric hospitalisation could contribute to the personalisation of care and the further development of IHT. It could also help to refine triage in patients undergoing treatment for an acute psychiatric crisis.

To our knowledge, there have been only a few IHT cohort studies in the last decade presenting data about factors associated with hospitalisation. Factors that have repeatedly been found to be associated with more hospital admissions include: older age (11, 12), severity of psychotic symptoms (11, 12), deliberate self-harm (11, 13, 14), previous psychiatric hospitalisation (11, 13, 14), and referral from accident and emergency departments (14) or the police (11). Factors have found to be associated with more hospital admissions include: being male (14), unemployed (8), unmarried (12), not being amenable to assessment (14), history of compulsory admission (14), diagnosed with non-affective psychotic disorders (12), and contact or support from community mental health centres within 48 h prior to admission (11).

A shared feature of these studies is that they evaluate the predictive validity of sometimes large numbers of seemingly related constructs without clustering them in overarching domains. Hence, there are no studies that clustered putative risk factors into categories or domains and studied the relationship between such domains and psychiatric hospitalisation of patients treated for an acute psychiatric crisis who had access to IHT. Therefore, in order to paint a clearer picture of the interrelatedness of the individual risk factors, our study first distinguished three main categories of predictors: (1) Individual and environment, (2) Psychopathology and care and (3) Quality of life. To refine and operationalise these broad categories, we divided these categories in hypothetical domains based on our clinical experience and on previous research (11, 13–16). Thus, conceptually related putative risk factors that have previously been shown to be associated with hospitalisation pursuant to treatment for a psychiatric crisis were clustered into seven overarching domains. This is done because we think that these overarching domains are more tangible in the context of making decisions about hospitalisation than individual predictors. Two domains, “sociodemographic characteristics” and “social engagement” comprise the category Individual and environment. Four domains, “diagnosis and psychopathology,” “aggression,” “substance use,” and “mental health services” comprise the category Psychopathology and care; the domain “quality of life” is placed in its own category.

Particularly the “aggression” domain is often presented in the literature as a predictor of psychiatric hospitalisation of IHT patients. Certain factors in this domain, such as not being amenable to assessment, violence during an episode of illness or referral by the judiciary, have repeatedly been found to be positively associated with hospitalisation (7, 11, 14). We therefore expected the “aggression” domain to explain the highest percentage of variance in hospitalisation and that individual factors in this domain would continue to be positively associated with hospitalisation, even after adjustment for the other domains. This implies that patients in this domain will have a higher relative and absolute risk of hospitalisation than those who are not (17, 18).

The aim of this study is to identify factors associated with hospitalisation within 6 weeks after a psychiatric crisis in patients allocated to the IHT-arm of a large randomised controlled trial. The 6 weeks follow-up period was chosen based on the duration of the intervention period of IHT and a previous randomised controlled trial (RCT) (1, 6). We were interested in identifying factors that predict hospitalisation during the IHT intervention. A secondary aim is to identify the most predictive factors in the seven domains that are associated with hospitalisation, using both relative and absolute measures of risk. What this study adds is that it determines risk factors and domains associated with psychiatric hospitalisation of patients treated for an acute psychiatric crisis who had access to IHT.

## Methods

This study draws on data acquired during a RCT assessing the effects of IHT in comparison to Care as Usual (CAU). The study protocol, including design and rationale of the RCT and screening and recruitment of the patients, has been presented elsewhere (6). The Medical Ethics Committee of VU University Amsterdam (METc VUmc) approved the study (#NL55432.029.16) and the trial was registered in the Netherlands (#NTR6151). This study complies with the ethical standards laid down in the 1964 Declaration of Helsinki and its later amendments.

### Recruitment and Inclusion Criteria

Patients were enrolled from two mental health organisations that provide high-intensity psychiatric care through IHT or hospital admission. Patients were recruited by IHT teams and from psychiatric wards between November 2016 and October 2018. Patients included in the RCT were between 18 and 65 years of age; experiencing an acute psychiatric crisis severe enough to warrant hospital admission by a psychiatrist; classified according to the DSM-IV-TR or DSM-V with at least one axis I or II disorder; and residents of Amsterdam, the Netherlands. Patients were excluded if they: were homeless, had a primary classification of substance use disorder or intellectual disabilities; lacked basic knowledge of the Dutch language; received (Flexible) Assertive Community Treatment care [(F)ACT] (19); or had previously received IHT. Patients who receive (F)ACT care were excluded because they already receive ongoing care. Moreover, (F)ACT-teams have the option to quickly up-scale their care by providing outreach work and more frequent house visits when the patient's condition deteriorates. IHT provides care for patients that have no community care. There were no other inclusion or exclusion criteria.

### Design, Pre-randomisation, and Data Collection

The two-arm Zelen double consent design (20) was used for the randomised controlled IHT study. Patients who met the study criteria were pre-randomised to IHT or CAU, and an allocation ratio of 2:1 was applied for reasons of staff and facility capacity. Before participation, there was an assessment of the capacity to consent to research participation and mentally incompetent patients were excluded. After patients gave written informed consent, they underwent initial interviews by trained researchers with follow-up interviews at 6, 26, and 52 weeks post-randomisation. The study sample for the present study consisted of the patients included in the RCT who were allocated to the experimental group (IHT). In addition, the present study analysed only the baseline and 6 weeks follow-up measurements. The 6 weeks follow-up period was selected as it marks the end of the intervention period of IHT in or as well as in a previous RCT (1, 6). We were interested in identifying risk factors that predict hospitalisation during the IHT intervention.

All collected data were stored for at least 15 years after the last participant's interview. Storage of data was supervised by the principal investigator and complies with the Dutch Personal Data Protection Act.

### Intervention

As stated elsewhere (5, 6), IHT is an intensive short-term outpatient care modality which provides intensive care more than twice a week and continues for an average of 6 weeks until a crisis is resolved. IHT is provided by multidisciplinary teams who act as gatekeepers for psychiatric hospitalisation by assessing every patient and taking into consideration the necessity of hospitalisation and the outlook with IHT. IHT teams can provide care in two ways: (1) immediately before hospitalisation; or (2) as soon as discharge is being considered. For patients who were hospitalised after IHT was initiated because of the severity of their crisis, IHT could be resumed after discharge.

### Outcome Measure

Participants allocated to IHT who were hospitalised in the first 6 weeks after inclusion in the study were labelled “hospitalised”. These participants were admitted to a psychiatric ward despite being offered IHT, indicating that prevention of hospitalisation was not successful. Participants allocated to IHT who received only outpatient care during these 6 weeks were labelled “non-hospitalised”.

### Predictors

As stated above, to provide a better overview of the interrelatedness of the various constructs in our analysis, we allocated our predictors to seven domains that were clustered in three main categories: “individual and environment,” “psychopathology and care,” and “quality of life”. The categories, domains and factors included were as follows:

#### Individual and Environment

##### Sociodemographic

The baseline interview measured the following: age, gender (female/male), country of birth (Netherlands/other), educational level categorised using the education mapping standards (low/middle/high) (21), employment status (unemployed/employed), family net income (low income <1,500 euros a month/high income >1,500 euros a month).

##### Social Engagement

A self-developed questionnaire was used to assess patient perceptions of social support (on a five-point Likert-type scale with higher scores indicating more social support) and network extent (up to five persons). The self-report Trimbos Institute and Institute of Medical Technology Questionnaire for Costs Associated with Psychiatric Illness (TiC-P) (22) was also used to determine whether patients received any kind of social support. The extent of the social network was measured by asking about patients' domestic situation (living alone/living with others) and marital status (single, divorced or widowed/relationship). Additionally, the Health of the Nation Outcome Scales (HoNOS) was used to assess problems with relationships and social isolation (item 9) and problems with living conditions (item 11) (23).

#### Psychopathology and Care

##### Diagnosis and Psychopathology

Psychiatric disorders were classified by a psychiatrist using the Diagnostic and Statistical Manual of Mental Disorders (DSM-IV-TR or DSM-V depending on the year of inclusion) (24). Self-injury was measured using the HoNOS (item 2). The Brief Symptom Inventory (BSI) (25) and the Brief Psychiatric Rating Scale (BPRS) (26, 27) were used as self-reported questionnaires to measure the psychopathology of the participant.

##### Aggression

Health care professionals measured overactive, aggressive, disruptive or agitated behaviour by completing the HoNOS (item 1) at the outset of treatment. Contact with police or the judiciary in the 6 months prior to the initiated crisis was assessed.

##### Substance Use

Two questionnaires were included in this domain that demonstrate substance use which is considered to be a proxy to substance use disorder, a form of psychopathology. Alcohol use in the previous 12 months was measured using the Alcohol Use Disorder Identification Test (AUDIT) (28). This instrument screens for unhealthy alcohol use, defined as risky or hazardous consumption or any alcohol use disorder. Substance use in the previous 30 days was assessed with the Measurements in the Addictions for Triage and Evaluation (MATE Interview) (29). This questionnaire was designed to make a valid and reliable assessment of various patient characteristics for the purpose of referring patients to substance abuse treatment and evaluating the treatment that is provided.

##### Mental Health Services

The variables relating to the use of the health services in the previous year included: admission (yes/no), length of admission (days), compulsory admission (yes/no) and number of treatment sessions. This information was obtained from patients' digital health records. The number of recorded contacts with different health care professionals such as psychiatrists, nurses or general practitioners (GP) was measured with the TiC-P questionnaire and obtained from the digital health records administered by the two mental health organisations involved.

#### Quality of Life

The quality of life main category consists of one domain. The domain quality of life has been measured by two independent questionnaires. The European Quality of Life-5 Dimensions (EQ-5D-5L) (30) and the Manchester Short Assessment of Quality of Life (MANSA) (31). Both instruments have been extensively described in the literature as qualified questionnaires that measure generic health status.

### Data Analysis

Participants allocated to IHT were included in the analysis regardless of whether they received IHT or not (intention-to-treat). The characteristics of the patients included are presented here as frequencies and percentages or means with the standard deviations.

A three-step approach was used to estimate the explained variance in different domains associated with hospitalisation within 6 weeks after the onset of the psychiatric crisis. In the first step, the differences for all putative predictors between participants who were not hospitalised and those who were hospitalised were analysed using univariate analysis. Pearson's Chi-Square Test (categorical variables) or the Mann-Whitney *U* test were used in the univariate analysis for the non-log transformed variables. The Mann-Whitney *U* test was selected because almost none of the variables were normally distributed, even after log transformations. Secondly, variables with a *p* ≤ 0.05 were then assigned to domains and assessed for collinearity. In the case of domains with a variance inflation factor (VIF) higher than five, the least predictive variable was removed and VIF was checked again. If the VIF was still > 5, this process was repeated. In the third step, the domains were entered one at a time in the multiple logistic regression model. Nagelkerke pseudo *R*^2^ was used to state the explained variance for each of the seven domains. Moreover, the Nagelkerke pseudo *R*^2^ was assessed for all the domains together by entering all significant predictors from the first step in a single multiple logistic regression model.

A forward multiple logistic regression model was used to identify the most significant predictive variables in the domains associated with hospitalisation. The choice of this statistical approach was based on statistical methods used in a previous study (13). Moreover, the forward procedure limits a possible overfitting of the model (with the criterion of 10–15 variable per event) and is less susceptible to collinearity (32, 33). All significant predictive variables from the first step were entered in the forward multiple logistic regression model. The forward multiple logistic regression model starts with only an intercept and no predictors. At each step the variable not yet in the equation with the smallest probability in the *F*-test is entered, as long as the value is smaller than the cut-off value for inclusion in the model. We used the default inclusion cut-off value of SPSS (*p*_in_ = 0.05). Ultimately ending with the full model including all statistically significant predictive variables. Statistical tests for the forward multiple logistic regression model were performed with a significance level of *α* = 0.05.

The final forward multiple logistic regression model provided odds ratios with confidence intervals. To obtain relative risk ratios with confidence intervals we performed a Poisson regression including the variables of our final model. In addition, we obtained absolute risk probabilities and absolute risk differences with confidence intervals from a linear probability model (generalised linear models module SPSS). To obtain the RR and ARD for the HoNOS item 1 we clustered the scores 0 (“no problem”) and 1 (“minor problem”) and on the other hand the scores 2 (“mild problem”), 3 (“moderately severe problem”) and 4 (“severe to very severe problem”) (34). Determining relative and absolute risk establishes a clearer picture of the clinical significance of the risk factors that are identified. Furthermore, from the perspective of the patients, absolute risk often provides more relevant information since it expresses what patients can expect from IHT when they have one of the identified risk factors (35). All statistical analyses were performed using SPSS Statistics software version 26.0 for Windows.

## Results

### Study and Participant Characteristics

In the RCT that assesses the effects of IHT by comparison with CAU, 246 patients participated in the RCT study. Of the 246 participants, 183 were pre-randomised in the IHT-arm and 63 in the CAU-arm (Supplementary Figure 1). Of the 246 participants in total, the mean age was 41.01 (SD = 12.66) and there were 135 (54.9%) female participants. The analyses of this study were based on the data of 183 participants who were allocated to the IHT-arm. A total of 183 patients agreed to participate in the study, of whom 37 (20.2%) agreed to the use of their medical records only (no interviews). Within the first 6 weeks after allocation to IHT, 69 (37.7%) participants completed IHT without being hospitalised. Despite the fact that they had access to IHT teams, 114 (62.3%) patients were hospitalised within 6 weeks: 89 (78.1%) were hospitalised immediately and 25 (21.9%) received IHT but were still hospitalised during the intervention period. The analyses were based on the data of 183 participants.

Descriptive statistics for the study population and the differences between the non-hospitalised and hospitalised groups are shown in Table 1. Significantly more participants who were male, not employed and who were in the lower-income group were hospitalised within the first 6 weeks after allocation to IHT. By comparison with their non-hospitalised counterparts, participants who were hospitalised were significantly more aggressive, they had had more contact with the police or judiciary over the past 6 months. They had also used more cannabis in the past 30 days, and they had a better quality of life. Participants diagnosed with schizophrenia spectrum and other psychotic disorders represented the highest proportion of the study population and were hospitalised significantly more often. Participants who were hospitalised had more positive symptoms and were more disoriented (higher scores on the BPRS) in comparison to non-hospitalised participants.

**Table 1:** Characteristics of the Intensive home treatment cohort, comparison between non-hospitalised and hospitalised group.

**Factors**	**Total *N***	**All participants**	**Non-hospitalised**	**Hospitalised**	***p-*value**^**‡**^
**Sociodemographic**					
Age: mean (SD)	183	40.03 (12.71)	40.19 (12.09)	39.94 (13.12)	0.87
Gender: *n* (%), female	183	105 (57.4)	49 (71.0)	56 (49.1)	<0.01
Country of birth: *n* (%), the Netherlands	147	116 (78.9)	42 (76.4)	74 (80.4)	0.56
Education level	146				0.60
Low		13 (8.9)	5 (9.1)	8 (8.8)	
Middle		75 (51.4)	31 (56.4)	44 (48.4)	
High		58 (39.7)	19 (34.5)	39 (42.9)	
Employed: *n* (%), yes	146	75 (51.4)	36 (66.7)	39 (42.4)	<0.01
Income: *n* (%), high	137	67 (48.9)	34 (66.7)	33 (38.4)	<0.01
**Aggression**					
Agitated behaviour (HoNOS 1): mean (SD)	78	1.38 (1.24)	0.88 (0.96)	1.76 (1.30)	<0.01
Police or judiciary: *n* (%), yes	142	67 (47.2)	11 (21.6)	56 (61.5)	<0.001
**Diagnosis and psychopathology**					
Schizophrenia spectrum and other psychotic disorders: *n* (%)	183	59 (32.2)	12 (17.4)	47 (41.2)	<0.01
Depressive disorders: *n* (%)	183	41 (22.4)	23 (33.3)	18 (15.8)	<0.01
Bipolar Disorders: *n* (%)	183	33 (18.0)	8 (11.6)	25 (21.9)	0.08
Self-injury (HoNOS 2): mean (SD)	78	0.79 (1.17)	0.70 (0.95)	0.87 (1.31)	0.91
BSI total score: mean (SD)	131	1.11 (0.76)	1.34 (0.72)	0.99 (0.76)	0.01
BPRS total: mean (SD)	137	1.81 (0.41)	1.83 (0.38)	1.81(0.42)	0.52
Positive symptoms	137	1.81 (0.91)	1.60 (0.76)	1.92 (0.97)	0.03
Negative symptoms	137	1.26 (0.33)	1.29 (0.30)	1.24 (0.34)	0.15
Depression and anxiety symptoms	137	2.58 (1.03)	3.04 (0.91)	2.34 (1.01)	<0.001
Disorganisation	137	1.61 (0.53)	1.46 (0.37)	1.68 (0.58)	0.03
**Quality of life**					
EQ-5D-5L: mean (SD)	137	0.77 (0.24)	0.71 (0.28)	0.80 (0.22)	0.03
MANSA total: mean (SD)	131	4.84 (1.19)	4.66 (1.07)	4.92 (1.24)	0.13
**Substance use**					
Alcohol use: mean (SD)	139	5.45 (6.09)	4.98 (5.62)	5.70 (6.34)	0.56
Cannabis: *n* (%), yes	143	39 (27.3)	5 (9.6)	34 (37.4)	<0.001
Other: *n* (%), yes	147	12 (8.2)	3 (5.5)	9 (9.8)	0.35
**Social engagement**					
Domestic situation: *n* (%), living alone	145	54 (37.2)	18 (33.3)	36 (39.6)	0.45
Marital status: *n* (%), single, divorced or widowed	146	94 (64.4)	31 (57.4)	63 (68.5)	0.18
Size social network: mean (SD)	147	3.95 (1.53)	4.39 (1.10)	4.15 (1.16)	0.62
Social network support: mean (SD)	137	4.40 (0.68)	4.35 (0.65)	4.43 (0.70)	0.39
Social support received: *n* (%), yes	137	83 (60.6)	37 (74.0)	46 (52.9)	0.02
Relationships and social isolation (HoNOS 9): mean (SD)	78	1.27 (1.09)	1.15 (1.33)	1.36 (0.88)	0.14
Problems with living conditions (HONOS 11): mean (SD)	78	0.58 (1.08)	0.45 (1.09)	0.67 (1.09)	0.14
**Mental health services**^**§**^					
Admission: *n* (%) yes	183	18 (9.8)	5 (7.2)	13 (11.4)	0.36
Length of admission: mean (SD)	183	2.13 (13.29)	0.74 (3.93)	2.96 (16.53)	0.36
Compulsory admission: *n* (%), yes	183	6 (3.3)	0 (0.0)	6 (5.3)	0.05
General practitioner: mean (SD)^†^	142	1.97 (2.92)	2.61 (2.66)	1.62 (3.01)	<0.01
Specialised nurse: mean (SD)	142	5.51 (8.43)	5.76 (9.58)	5.37 (7.77)	0.95
Psychologist/ Psychiatrist: mean (SD)	142	11.50 (24.78)	11.60 (17.84)	11.44 (28.02)	0.63
Other mental health care professionals: mean (SD)	142	2.02 (3.85)	2.14 (3.46)	1.95 (4.07)	0.51
Treatment sessions: mean (SD)	183	8.57 (18.91)	9.94 (19.55)	7.74 (18.55)	0.49

† *Number of contacts with the general practitioner during the previous 3 months*.

‡ *Pearson's Chi-Square Test was used for the categorical variables and the Mann-Whitney U test was used for the continuous variables*.

§ *Mental health service use during the previous year*.

Significantly more participants had received social support within the previous 3 months in the non-hospitalised group. Our data also show that all participants with a history of compulsory admission were hospitalised within 6 weeks after the onset of the psychiatric crisis. Patients in the hospitalised group had significantly fewer contacts with their GP in the year before the initial crisis than patients in the non-hospitalised group.

### Explained Variance in the Domains

Explained variances in the different domains associated with hospitalisation are shown in Table 2. The highest explained variance was found for the predictive factors in the “aggression” domain (pseudo *R*^2^ = 0.34). This domain consist of two predictive factors, agitated behaviour (OR = 2.13 with 95% CI = 1.06–4.28, *p* = 0.03) and contact with police or judiciary (OR = 6.60 with 95% CI = 1.75–24.85, *p* = 0.01). Both predictive factors were significantly associated with hospitalisation. The “diagnosis and psychopathology” domain accounted for 0.28 of the explained variances in hospitalisation. This domain consists of six predictive factors. Five out of six factors showed a non-significant association with hospitalisation (*p* ≥ 0.05). Only participants diagnosed with schizophrenia spectrum and other psychotic disorders had higher odds to be hospitalised compared to participants with another diagnosis (OR = 3.82 with 95% CI = 1.19–12.25, *p* = 0.02). In the “sociodemographic” domain (pseudo *R*^2^ = 0.16) two of the three factors were significantly associated with hospitalisation. Being male was positively associated with hospitalisation (OR = 2.29 with 95% CI = 1.07–4.91, *p* = 0.03), while having higher income was negatively associated with hospitalisation (OR = 0.40 with 95% CI = 0.18–0.90, *p* = 0.03). In the “substance (ab)use” domain (pseudo *R*^2^ = 0.13), the factor cannabis use was significantly associated with hospitalisation (OR = 5.61 with 95% CI = 2.03–15.47, *p* < 0.01). The “mental health services” domain accounted for 0.08 of the explained variance in hospitalisation. This domain consist of two predictive factors, compulsory admission (OR = 872561898 with 95% CI = 0.00 -., *p* = 1.00) and general practitioner (OR = 0.89 with 95% CI = 0.78–1.02, *p* = 0.09). Both factors, compulsory admission and general practitioner factors were not independently associated with hospitalisation. The “social engagement” domain accounted for 0.06 of the explained variance in hospitalisation. This domain consist of one predictive factors, social support received (OR = 0.39 with 95% CI = 0.18–0.84, *p* = 0.02) was significantly associated with hospitalisation. Furthermore, 0.05 of the variance of hospitalisation was explained by the factors in the “quality of life” domain. The EQ-5D-5L was significantly associated with hospitalisation OR = 4.79 with 95% CI = 1.11–20.59, *p* = 0.04).

**Table 2:** Explained variance in the different domains related to hospitalisation.

**Factors per domain**	**Odds ratio**	**95% CI for odds ratio**		***p-*value**
		**Lower**	**Upper**	
**Sociodemographic (*****n*** **=** **137)**				
Intercept	2.84			<0.01
Gender	2.29	1.07	4.91	0.03
Employed	0.52	0.23	1.18	0.12
Income	0.40	0.18	0.90	0.03
*Nagelkerke pseudo R^2^: 0.16*				
**Aggression (*****n*** **=** **60)**				
Intercept	0.33			0.05
Agitated behaviour (HoNOS 1)	2.13	1.06	4.28	0.03
Police or judiciary	6.60	1.75	24.85	0.01
*Nagelkerke pseudo R^2^: 0.34*				
**Diagnosis and psychopathology (*****n*** **=** **131)**				
Intercept	0.93			0.95
Schizophrenia spectrum and other psychotic disorders	3.82	1.19	12.25	0.02
Depressive disorders	0.56	0.21	1.53	0.26
BSI total score	1.01	0.49	2.08	0.97
Positive symptoms	0.87	0.48	1.55	0.63
Depression and anxiety symptoms	0.68	0.39	1.18	0.17
Disorganisation (BPRS)	3.22	0.98	10.62	0.05
*Nagelkerke pseudo R^2^: 0.28*				
**Quality of life (*****n*** **=** **137)**				
Intercept	0.58			0.36
EQ-5D-5L	4.79	1.11	20.59	0.04
*Nagelkerke pseudo R^2^: 0.05*				
**Substance use (*****n*** **=** **143)**				
Intercept	1.21			0.33
Cannabis	5.61	2.03	15.47	<0.01
*Nagelkerke pseudo R^2^: 0.13*				
**Social engagement (*****n*** **=** **137)**				
Intercept	3.15			<0.001
Social support received	0.39	0.18	0.84	0.02
*Nagelkerke pseudo R^2^: 0.06*				
**Mental health services (*****n*** **=** **142)**				
Intercept	2.14			<0.01
Compulsory admission	872561898.85	0.00		1.00
General practitioner	0.89	0.78	1.02	0.09
*Nagelkerke pseudo R^2^: 0.08*				

The model with all the domains included 16 variables and those variables together accounted for 75% (pseudo *R*^2^ = 0.75) of the explained variance in hospitalisation and a good fit to the data as determined by the Hosmer-Lemeshow test (chi-square = 2.44, *p* = 0.97) (see Supplementary Material).

### Risk of the “Aggression” Domain

The strength of the association between the “aggression” domain and hospitalisation was assessed by entering the variables based on the explained variance in the forward multiple logistic regression model. As shown in Table 3, a strong association with hospitalisation was found to persist for three of the seven domains.

**Table 3:** Potential predictors associated with hospitalisation during 6 weeks follow-up.

**Factors**	**B**	**SE**	**Wald**	***p-value***	**OR**	**95% CI for OR**		**RR**	**95% CI for RR**		**ARD**	**95% CI for ARD**	
						**Lower**	**Upper**		**Lower**	**Upper**		**Lower**	**Upper**
Intercept	−0.22	0.87	0.07	0.80	0.80								
Judiciary and police	2.02	0.98	4.25	0.04	7.55	1.10	51.63	1.47	0.70	3.12	0.24	0.03	0.46
Agitated behaviour (HoNOS 1)	1.03	0.52	3.99	0.05	2.80	1.02	7.72	1.36^*^	0.65	2.83	0.22^*^	0.00	0.44
Schizophrenia spectrum and other psychotic disorders	3.10	1.30	5.68	0.02	22.22	1.74	284.54	1.50	0.73	3.11	0.31	0.09	0.53
Employment	−2.35	0.96	5.99	0.01	0.10	0.01	0.63	0.66	0.33	1.33	−0.28	−0.48	−0.07

*Outcome of forward multiple logistic regression including risk ratio, relative and absolute risk measures*.

*Total number of participants included in the analysis was 51*.

*Degrees of freedom: 1. OR = Odds ratio. RR = Relative risk. ARD = Absolute risk difference*.

* *To compute the RR and ARD for the HoNOS 1, the cluster of scores 0 and 1 were compared to the cluster of scores 2, 3, and 4*.

From the “aggression” domain, two predictive factors were associated with hospitalisation. Participants who had been in contact with the police or judiciary during the 6 months before the crisis were more likely to be hospitalised within 6 weeks after the onset of the initial crisis (OR = 7.55 with 95% CI = 1.10–51.63, *p* = 0.04). The absolute risk for participants who had been in contact with the police or judiciary was 0.76, as opposed to 0.52 for those who had not been in contact with the police or judiciary. This means that 24% of the risk of being hospitalised was specifically attributable to this factor. A relative risk of 1.47 was found for this factor, indicating that the risk of hospitalisation was 47% higher for patients who had been in contact with the police or judiciary than for patients who had not.

Participants who had a higher score on the HoNOS 1, indicating more agitated behaviour, were more likely to be hospitalised within 6 weeks after the onset of the initial crisis (OR = 2.80 with 95% CI = 1.02–7.72, *p* = 0.05). The absolute risk for participants with mild to severe agitated behaviour (HoNOS 1: score 2, 3, and 4) was 0.74, as opposed to 0.52 for those who showed no to minor (HoNOS 1: score 0 and 1) agitated behaviour at the outset of the treatment. This means that 22% of the risk of being hospitalised was specifically attributable to mild to severe agitated behaviour. A relative risk of 1.36 was found for this factor, indicating that the risk of hospitalisation was 36% higher for patients who showed mild to severe agitated behaviour.

From the “diagnosis and psychopathology” domain, one predictive factor was positively associated with hospitalisation. Participants who were diagnosed with schizophrenia spectrum and other psychotic disorders were more likely to be hospitalised within 6 weeks after the onset of the initial crisis (OR = 22.22 with 95% CI = 1.74–284.54, *p* = 0.02). The absolute risk for participants who have been diagnosed with schizophrenia spectrum and other psychotic disorders was 0.83, as opposed to 0.52 for those who had another diagnosis. This means that 31% of the risk of being hospitalised was specifically attributable to this factor. A relative risk of 1.50 was found for this factor, indicating that the risk of hospitalisation was 50% higher for patients who were diagnosed with schizophrenia spectrum and other psychotic disorders compared to patients who had another diagnosis.

From the “sociodemographic” domain, only employment status was associated with hospitalisation. However, this factor showed a negative association with hospitalisation, indicating that participants who were employed before the crisis were less likely to be hospitalised within 6 weeks after the onset of the initial crisis (OR = 0.10 with 95% CI = 0.01–0.63, *p* = 0.01). The absolute risk for participants who were employed was 0.24, as opposed to 0.52 for those who were not employed. This means that there is an absolute risk difference of 28%. A relative risk of 0.66 was found for this factor, indicating that the risk of hospitalisation was 34% lower for participants who were employed compared to those participants who were unemployed.

The presented model gave an explained variance of 0.62 and had a good fit to the data as determined by the Hosmer-Lemeshow test (chi-square = 2.43, *p* = 0.97).

## Discussion

The aim of the present study was to determine the explained variances in different domains associated with hospitalisation of patients from psychiatric emergency services with access to IHT. In the present study, 16 out of 38 putative risk factors were associated with hospitalisation. The “aggression” and “diagnosis and psychopathology” domains accounted for the highest proportion of explained variance. Furthermore, we hypothesised that the “aggression” domain would be closely associated with psychiatric hospitalisation. In our assessment of this hypothesis, even after forward multiple logistic regression, the factor contact with the police or judiciary and agitated behaviour remained positively associated with hospitalisation. Our hypothesis was therefore confirmed, indicating that patients fulfilling this criteria are more likely and have a higher risk to be hospitalised.

Our findings are in line with previous studies that have shown that risk factors in the “aggression” domain are associated with hospitalisation (11, 14, 16, 36). The present study indicates that patients with a history of encounters with the police or judiciary have an additional risk of 24% of being hospitalised. Previous contact with the police or judiciary, especially police involvement during the admission process, could be an indication of aggressive behaviour or a complex clinical condition that lead to a higher risk of hospitalisation. Our findings showed that the relative risk for hospitalisation was 47% higher for patients who had been in contact with the police or judiciary than for patients who had not. In addition, patients who were more overactive, aggressive, disruptive or agitated (score ≥ 2) had 36% higher risk of hospitalisation compared to those with no to minor overactive, aggressive, disruptive or agitated behaviour. A decision to admit a patient is often based on the urgency of treatment given the psychopathology and the risk of harm to the patient or others since these two factors are admission criteria under the 1983 Dutch Mental Health Act. This implies that these patients are less likely to complete IHT without hospitalisation within 6 weeks.

The “diagnosis and psychopathology” domain explained 28% of the variance in hospitalisation. The risk factor schizophrenia spectrum and other psychotic disorders was found to be positively associated with hospitalisation, meaning that patients with this diagnosis were less likely to complete IHT without being hospitalised. Even in the forward multiple logistic model, a strong positive association persisted between schizophrenia spectrum and other psychotic disorders and hospitalisation and the relative risk and absolute risk of hospitalisation were high. In line with our findings, a diagnosis of schizophrenia spectrum and other psychotic disorder has consistently been found to predict hospitalisation (12, 14, 16), whereas patients diagnosed with depression are less likely to be hospitalised (11, 12, 14).

The “sociodemographic” domain accounted for 16% of the explained variances in hospitalisation. Nonetheless, in the forward multiple logistic model, the factor employment status was negatively associated with hospitalisation. The relative risk and absolute risk of hospitalisation within 6 weeks were lower for those employed participants compared to unemployed. Having higher income or paid employment was found to be associated with preventing hospitalisation, which is in line with the previous findings in the literature (7, 8). The evidence is mixed regarding gender as a predictive factor of hospitalisation. Previous IHT cohort studies have found no relationship between gender and hospitalisation, contrary to our findings (8, 11–13). However, there are other studies that were in line with our findings, concluding that male patients have a higher risk of being hospitalised (14, 37). For the purposes of the further development of IHT and the refinement of triage for patients treated for an acute psychiatric crisis, health care professionals should pay more attention to unemployed patients and those diagnosed with schizophrenia spectrum and other psychotic disorder, since these patients benefit less from IHT in terms of the prevention of hospitalisation.

The “mental health services” domain included only two factors associated with hospitalisation and the explained variance was low. Surprisingly, and by contrast with other studies, there was no difference between patients who received only IHT and hospitalised patients in terms of previous admissions (11, 14). Our sample included only 14 (out of 183) patients with an admission history. We therefore lacked the statistical power to establish a correlation in this respect. The difference in the results relating to the history of the admission risk factor could be explained by the inclusion criteria used in the present study since we excluded patients receiving (F)ACT. The patients who receive (F)ACT are severely ill and they have complex needs and often require psychiatric inpatient care (38). In the present study, this history of the low utilisation of health services could indicate that our patient population had been introduced to the mental health system recently or had been more stable in previous years. However, we do not have specific data to support this assumption.

The association between psychiatric hospitalisation and contacts with a health professional has not been studied previously in this patient population. Our results show a trend toward a negative association between repeated visits to a GP and hospitalisation (not significant). To understand this result better, two important considerations should be taken into account. Firstly, repeated visits may imply that patients are being monitored for relapse and that psychiatric crises are therefore identified sooner, preventing hospitalisation. Secondly, we assume that patients referred from GPs do not have long-term chronic mental health problems, that they are not yet using specialised mental health services, or that they suffer only incidental relapse. This assumption is confirmed by our results showing that visits to a psychiatrist or psychologist and the number of treatments by a psychiatrist or psychologist was not high in the previous year (7, 8, 11–14, 37, 39).

In this study, we assessed the portion of explained variance of quality of life. Although the domain “quality of life” presented a low portion of the explained variance in hospitalisation, the individual factor showed that participants with a higher quality of life scores were more often hospitalised. The result of patients with a higher quality of life scores who were more often hospitalised could be related to the type of diagnosis. Patients with depression (39) or anxiety symptoms had a lower quality of life scores and were less often hospitalised. Participants with bipolar or schizophrenic disorders (40, 41), were more often hospitalised and would probably score higher on the quality of life scores list (for example due to mania). A recent study by Elegbede et al. showed a slightly higher quality of life toward hospitalisation (not significant) (42). The hypothesis about the relationship between the type of mental health disorders and quality of life should be evaluated by future research.

We also found that cannabis use during the past 30 days, a risk factor included in the “substance use” domain, was positively associated with hospitalisation. This means that patients who used cannabis in the 30 days before the initial crisis were less likely to complete IHT without being hospitalised. However, previous studies assessing factors relating to hospitalisation, specifically from psychiatric emergency departments, have found that substance use is associated with a lower probability of hospitalisation. Those studies concluded that a crisis induced by substance use can be resolved during a relatively short stay in the emergency unit (40, 41). Furthermore, Hasselberg et al. reported no differences relating to substance abuse between patients who received IHT and hospitalised patients (11). The differences between our findings and previous studies could be explained by variations in the timing of the assessment of substance use. The studies referred to assessed substance use when the patients were hospitalised, whereas the present study asked patients about substance use during the previous 30 days. A history of cannabis use could reflect an underlying problem that leads to a psychiatric crisis.

Unexpectedly, the “social engagement” domain accounted for only 6.0% of the explained variance in hospitalisation. This was unexpected because this domain is a fundamental construct in the IHT model. By contrast with previous studies, we did not find an association between hospitalisation and the putative factors “social network size” and “degree of support” (42, 43). Nevertheless, we found that patients who received social support during a crisis were less likely to be hospitalised. Apparently, social support from a patient's social network is a stronger predictor for non-hospitalisation within 6 weeks after the onset of the initial crisis than the size of a patient's social network of the patient or the amount of support from the social network. Regular contact with a patients' social network that can identify the potential triggers of a crisis reduces the probability of a relapse. IHT teams should continue to encourage the involvement of a patient's social network in care.

### Strengths and Limitations

An important strength of this study is the inclusion of a large proportion of patients that were offered IHT at the onset of an acute psychiatric crisis. The psychosocial variables used for the analyses reflect realistic, pragmatic and clinical practise. Another strength is that this study was part of an RCT, which means that patients were randomly allocated to IHT and the data were analysed in line with the intention-to-treat principle. Additionally, the data used were obtained from two specialised mental health care organisations in Amsterdam that cover more than 80% of the demand for, and treatment of, mental health care in a large urban setting. It is therefore likely that our findings can be generalised to other urban settings in similar patient populations.

Some possible limitations should be considered. Firstly, variables relating to the organisation of mental health services, such as the availability of beds and the characteristics of the IHT teams, including openings times and waiting lists, were unfortunately not considered for their effect. Secondly, the baseline measurement was conducted on average 3 weeks after the onset of the psychiatric crisis and it could be argued that this delay is too long, precluding the accurate measurement of the severity of the psychiatric crisis. However, earlier assessment was not feasible as the research group had to ensure that patients had the mental capacity to participate in the study. Thirdly, the generalisability of the findings is a factor that should be considered since our study population excluded patients with chronic mental health illness that already received community care and also did not include patients from rural areas. Fourthly, the results were based on the 6 weeks follow-up of the IHT intervention period as described in the study design of the RCT used in this study. Long term predictive factors and domains associated with hospitalisation should be studied. Finally, the results of the forward multiple logistic regression model were based on a small proportion (*n* = 51) of participants, therefore the results should be interpreted with caution. The reduction of the included participants in this forward multiple logistic regression model was mainly caused by the HoNOS 1. This instrument was administered by health care professionals and future research should focus more on collecting data about aggression.

## Conclusions

IHT is a community treatment focusing on crisis management and the reduction of hospitalisation. Professionals working in crisis care should be more aware of the different domains and predictive factors found in this study so that they can identify patients who require more attention in order to prevent hospitalisation. Awareness should be raised among IHT teams and other professionals working in crisis care of the importance of previous involvement with the police or judiciary, aggressive behaviour at outset of treatment, the diagnosis of schizophrenia spectrum and other psychotic disorders and employment status. This information could contribute to more personalised care and the prevention of hospital admissions. Furthermore, patients diagnosed with depressive disorders seem to benefit from IHT in terms of preventing hospitalisation, whereas patients with psychotic disorders are actually more likely to be hospitalised. The results of this study are based on a patient population that was new to the mental health system. Future studies on predictive factors for IHT should include patients with chronic mental health illness that already received community care and patients from rural areas. Moreover, more patients' information would be interesting especially in the case of the “social engagement” domain.

## Data Availability Statement

The datasets generated for this article are not readily available because the datasets contain information that could compromise the privacy of research participants. Requests to access the datasets should be directed to Ansam Barakat, ansam.barakat@arkin.nl.

## Ethics Statement

The studies involving human participants were reviewed and approved by The Medical Ethics Committee of VU University Amsterdam (#NL55432.029.16) and the trial was registered in the Netherlands (#NTR6151). The patients/participants provided their written informed consent to participate in this study.

## Author Contributions

All authors contributed to the study conception and design. Material preparation and data collection were performed by AB and NML. Formal analysis and investigation were performed by AB and MB. The first draught of the manuscript was written by AB. All authors commented on previous versions of the manuscript. All authors read and approved the final manuscript.

## Conflict of Interest

The authors declare that the research was conducted in the absence of any commercial or financial relationships that could be construed as a potential conflict of interest.
