# Supplementary figures and images for: Police Encounters, Agitation, Diagnosis, and Employment Predict Psychiatric Hospitalisation of Intensive Home Treatment Patients During a Psychiatric Crisis
Source: Front Psychiatry. 2021 Feb 5;12:602912. doi: 10.3389/fpsyt.2021.602912 (PMC7901988; doi:10.3389/fpsyt.2021.602912)

Fig. 1 CONSORT flow diagram

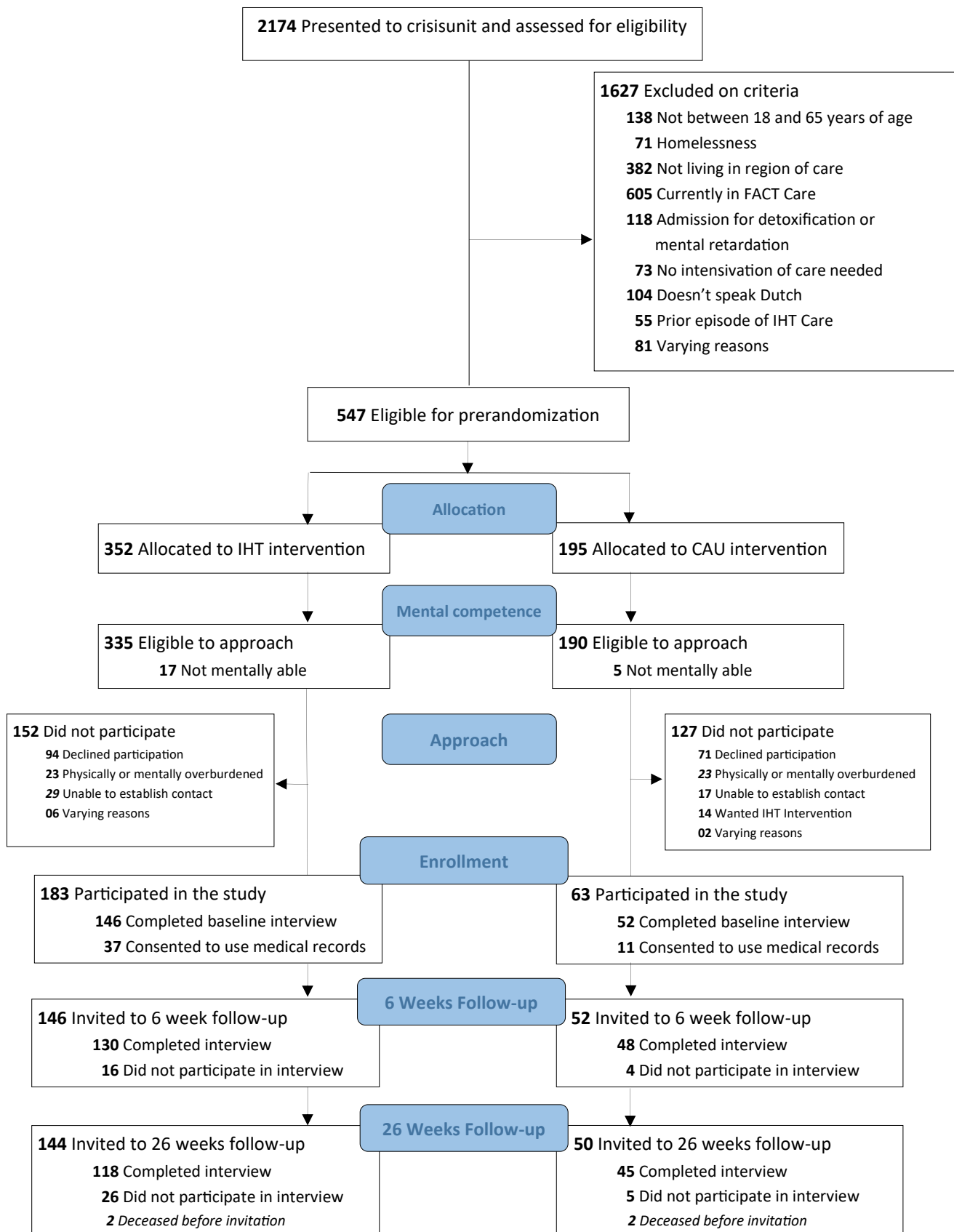

Supplement: Supplementary file 1 [file Image_1.PDF]
