# Supplementary material for: Police Encounters, Agitation, Diagnosis, and Employment Predict Psychiatric Hospitalisation of Intensive Home Treatment Patients During a Psychiatric Crisis
Source: Front Psychiatry. 2021 Feb 5;12:602912. doi: 10.3389/fpsyt.2021.602912 (PMC7901988; doi:10.3389/fpsyt.2021.602912)
Supplement: Supplementary file 2 [file Table_1.pdf]

Police Encounters, Agitation, Diagnosis and Employment Predict Psychiatric Hospitalisation of Intensive Home Treatment Patients During a Psychiatric Crisis

Ansam Barakat, Matthijs Blankers, Jurgen E. Cornelis, Louk van der Post, Nick M. Lommerse, Aartjan T. F. Beekman and Jack J. M. Dekker

Supporting Information: outcome of the multiple regression analysis

Factors	B	SE	Wald	*p*-value	Odds ratio	95% CI for odds ratio	
						Lower	Upper
Gender	-5.43	3.69	2.16	0.14	0.00	0.00	6.10
Employed	-4.63	2.80	2.74	0.10	0.01	0.00	2.35
Income	0.89	1.83	0.23	0.63	2.43	0.07	87.65
Aggression (HoNOS 1)	1.47	1.05	1.95	0.16	4.35	0.55	34.19
Disorganisation (BPRS)	0.42	2.38	0.03	0.86	1.52	0.01	162.23
Police or judiciary	6.88	4.41	2.43	0.12	967.89	0.17	5493617.08
Social support	-2.20	2.06	1.14	0.29	0.11	0.00	6.29
Depressive disorders	5.70	3.79	2.26	0.13	298.06	0.18	503431.13
Schizophrenia Spectrum and Other Psychotic Disorders	3.72	3.25	1.32	0.25	41.45	0.07	24098.30
BSI total score	-0.51	1.54	0.11	0.74	0.60	0.03	12.35
Positive symptoms	2.25	1.75	1.64	0.20	9.46	0.30	294.17
Depression and anxiety symptoms	1.22	1.19	1.04	0.31	3.38	0.33	35.11
Compulsory admission	21.97	13149.70	0.00	1.00	3470162450.74	0.00	.
General practitioner	-0.99	0.68	2.09	0.15	0.37	0.10	1.42
EQ-5D	0.24	4.26	0.00	0.96	1.27	0.00	5393.25
Cannabis use	-0.37	1.77	0.04	0.84	0.69	0.02	22.32
Constant	-3.72	7.33	0.26	0.61	0.02		

The total number of participants included in this analysis was 51 due to the availability of HoNOS 1.
Degrees of freedom: 1
